# Charge-Transfer Excitations within Density Functional Theory: How Accurate Are the Most Recommended Approaches?

**DOI:** 10.1021/acs.jctc.1c01307

**Published:** 2022-02-24

**Authors:** Dávid Mester, Mihály Kállay

**Affiliations:** Department of Physical Chemistry and Materials Science, Faculty of Chemical Technology and Biotechnology, Budapest University of Technology and Economics, Műegyetem rkp. 3., H-1111 Budapest, Hungary

## Abstract

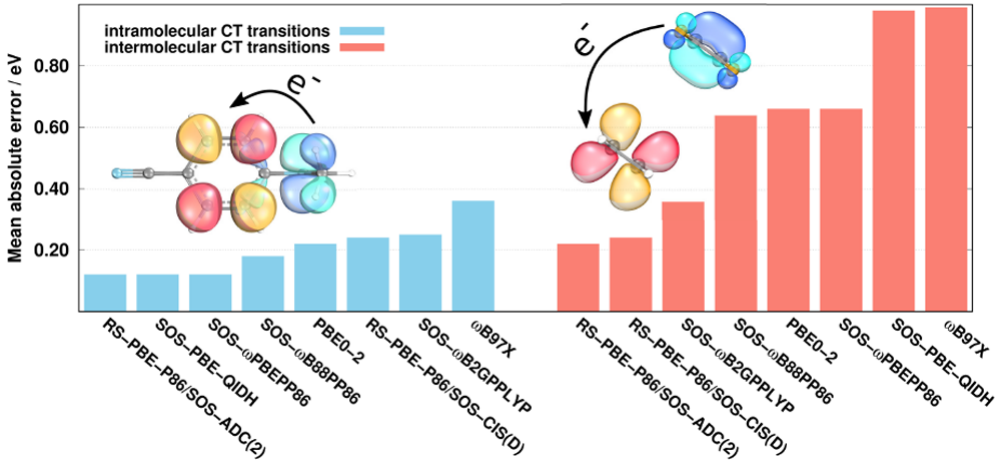

The performance of the most recent density functionals is assessed for charge-transfer (CT) excitations using comprehensive intra- and intermolecular CT benchmark sets with high-quality reference values. For this comparison, the state-of-the-art range-separated (RS) and long-range-corrected (LC) double hybrid (DH) approaches are selected, and global DH and LC hybrid functionals are also inspected. The correct long-range behavior of the exchange–correlation (XC) energy is extensively studied, and various CT descriptors are compared as well. Our results show that the most robust performance is attained by RS-PBE-P86/SOS-ADC(2), as it is suitable to describe both types of CT excitations with outstanding accuracy. Furthermore, concerning the intramolecular transitions, unexpectedly excellent results are obtained for most of the global DHs, but their limitations are also demonstrated for bimolecular complexes. Despite the outstanding performance of the LC-DH methods for common intramolecular excitations, serious deficiencies are pointed out for intermolecular CT transitions, and the wrong long-range behavior of the XC energy is revealed. The application of LC hybrids to such transitions is not recommended in any respect.

## 1 Introduction

Charge transfer (CT) transitions are cardinal phenomena in many areas of science. They play an important role in photovoltaics,^1^ where semiconducting materials, such as solar cells, convert the energy of light directly into electricity. The molecular conductance^2^ with single-molecule junctions is also described by CT states, and these transitions appear in biomolecular processes (e.g., molecular vision) as well.^3,4^ In addition, solvatochromism,^5−7^ one of the basic phenomena in photochemistry, is also related to the characteristic CT bands of transition metal complexes. These excitations are electronic transitions in which a large fraction of an electronic charge is transferred from one region, called the donor, to another, called the acceptor. In the case of intramolecular transitions, the donor and acceptor can be found on the same molecule, while intermolecular CT excitations take place between individual molecular entities. As distance plays a key role in the definition, it is easy to see that these states tend to appear in larger molecules. Accordingly, the development of reasonable computational methods to study such systems with proper accuracy is crucial.

Nowadays, time-dependent density functional theory (TDDFT)^8−11^ is the most popular choice to study time-dependent properties of extended molecular systems since its computational costs are relatively low. For semiquantitative accuracy, at least hybrid functionals are recommended, where the exchange–correlation (XC) energy contains Hartree–Fock contributions as well. While hybrid TDDFT excitation energies and spectral intensities are quite good for valence excitations, those for more challenging transitions, such as Rydberg states or excitations of extended π-electron systems, can still be qualitatively incorrect.^12,13^ This shortcoming, which originates from the wrong long-range behavior of the XC energy, also causes serious problems for CT transitions.^14−16^

To improve the results, several developments have appeared in the past two decades. One of the most notable directions is the double hybrid (DH) theory pioneered by Grimme.^17^ In this case, the XC energy is augmented with a second-order perturbation correction as well. In addition, higher-parametrized spin-scaled DH variants were also proposed,^18,19^ where the correction is replaced by the spin-component-scaled (SCS)^20^ or scaled-opposite-spin (SOS)^21^ second-order correlation energy. The excited-state analogues of such methods were elaborated by Grimme,^22^ Goerigk,^23^ and their co-workers. The improvements for excited-state properties were demonstrated in various excellent studies;^24−26^ however serious limitations were pointed out for CT transitions as well.^27−29^ An alternative solution can be the range-separated (RS) approaches. In this case, the Coulomb interaction is separated into long-range and short-range components.^30,31^ The related hybrid^32−38^ and DH analogues^39−42^ and their performances are well-known from the literature.^43−47^ The more approximate form of RS-DH theory, the family of the so-called long-range-corrected (LC) functionals, where solely the exchange contributions are range-separated,^18,48,49^ is also noteworthy. The excited-state variants of the RS-DH^29,50,51^ and LC-DH^27,52−54^ approaches were presented in the past years.

Benchmark calculations demonstrate that these methodological developments significantly improve the overall performance of the approaches.^26,50,53−55^ However, comprehensive studies focusing particularly on the challenging CT transitions cannot be found in the literature. On the one hand, as mentioned above, these state-of-the-art methods have been very recently published. On the other hand, extensive benchmark sets for CT excitations containing high-quality reference values have not been available to date. This problem was recently resolved by several authors. In this direction, one of the most promising attempts is the QUEST database created by Loos, Jacquemin, and co-workers,^56^ which contains different types of benchmark compilations. In their related contribution,^57^ the most popular intramolecular benchmark sets^52,58−61^ were collected, and new reference excitation energies relying on high-level ab initio calculations were suggested. Most of the molecules that were used for TDDFT benchmark studies in the last 15 years are included in this compilation. In addition, an intermolecular CT set using bimolecular complexes to ensure complete charge separation was recently proposed by Szalay and co-workers.^62^ For both compilations, the high-level reference values were calculated at the coupled cluster (CC) level including triple excitations, such as the CCSDT^63^ method and its approximate forms, namely, the CC3^64^ and CCSDT-3^65^ approaches. Furthermore, the reference values can be derived from experimental measurements as well.^66,67^ One of the most popular of these compilations was proposed by Baer and co-workers,^67^ and it has been used to demonstrate the performance of the LC-DH approaches.^27,28^

In this contribution, we compare the most advanced and robust TDDFT methods using comprehensive intra- and intermolecular CT benchmark sets. The correlation between the corresponding CT metrics and the errors in the excitation energies is discussed in detail, and the correct long-range behavior of the XC energy is also tested.

## 2 Charge-Transfer Metrics

The identification of CT excitations from the theoretical point of view often relies on subjective findings. To help with the unbiased characterization of such states, several descriptors were developed over the past decade.^58,68−72^ In the following, we briefly summarize two different approaches. The first one is easy to implement,^68^ while the second one is associated with the most recent purpose-designed program package.^73^

### 2.1 Orbital-Based Descriptors

The first orbital-based measure was proposed by Tozer and co-workers.^58^ In their approach, the overlap of the molecular orbitals (MOs) involved in the excitations was weighted as a function of the excitation coefficients. This metric has been successfully used as a diagnostic tool,^74^ but several deficiencies were pointed out for difficult cases.^75^ Later, the same formalism was applied by Guido et al.,^68^ but the overlap was replaced by the average distance of the hole–particle pair interactions. Accordingly, in their improved approach, the corresponding measure can be defined as
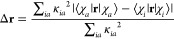
1where *i* and *a* are the occupied and virtual MO indices, respectively, and ⟨χ_*a*_|**r**|χ_*a*_⟩ is the corresponding MO (χ) centroid. As the Δ**r** index was originally developed for “full” TDDFT^8^ calculations, κ_*ia*_ denotes the sum of the corresponding coefficients of the excitation and de-excitation eigenvectors. Of course, it should be noted that the descriptor can be obtained within the Tamm–Dancoff approximation (TDA)^76^ as well. In that case, κ_*ia*_ simplifies to the single excitation coefficients.

In particular cases, dominant configurations cannot be identified in the wave function, which makes the characterization more difficult. A more compact representation of the excitations can be achieved via natural transition orbitals (NTOs).^77^ In this case, the one-particle transition density matrix (1TDM) is diagonalized, and the NTOs obtained can be sorted according to their importance. As shown in refs (78) and (79), it is advantageous to transform the canonical MO indices in eq 1 to the NTO basis. The resulting descriptor will be denoted by Δ**r**_NTO_.

### 2.2 Fragment-Based Descriptors

The elaboration of fragment-based metrics can be attributed to the pioneering works of Plasser and co-workers.^69,73,80−83^ In the case of such descriptors, the system is split up into different subspaces; let us denote them as *A*, *B*, etc. These units can be defined arbitrarily, but it is advantageous to invoke chemical intuition. The excitations are constructed by creating electron–hole pairs, and the separation of such pairs over the fragments is examined. The so-called charge transfer number from fragment *A* to fragment *B* can be defined as
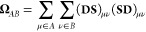
2where μ and ν are atomic orbital indices, **D** is the 1TDM, and the elements of the overlap matrix **S** are written as *S*_*μν*_ = ⟨χ_μ_|χ_ν_⟩. This measure can be interpreted as the probability of finding the hole on fragment *A* when the electron is on fragment *B* considering the atomic contributions to the individual fragments. The matrix **Ω** is usually used for graphical illustration of the hole and electron distributions via hole–particle correlation plots. In addition, a less abstract descriptor, the total amount of charge separation, is also calculated from the matrix elements of **Ω** as
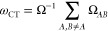
3where Ω = ∑_*AB*_Ω_*AB*_ is the normalization factor. The value of ω_CT_ ranges from 0 to 1, where the upper limit indicates complete charge separation.

To avoid a priori definition of the fragments, an additional measure was also proposed by Plasser and co-workers.^84^ The approximate exciton size is defined as
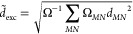
4where the charge transfer number is computed with respect to atoms *M* and *N* and *d*_*MN*_ is the distance between these atoms. This descriptor gives the root-mean-square separation of the electron and hole in a point charge approximation, which means that the spatial extent of the orbitals involved is neglected. Among many other measures, the descriptors discussed in this subsection are implemented in the highly recommended TheoDORE package.^73^

## 3 Computational Details

### 3.1 Calculation of the Numerical Results

All of the excitation energies were calculated using the Mrcc quantum-chemical program suite.^85,86^ The TDDFT calculations were carried out within the TDA. For the calculations, Dunning’s correlation consistent basis sets (cc-pV*X*Z, where X = D, T)^87,88^ were used. The density-fitting approximation was utilized for both the ground and excited states, and the corresponding auxiliary bases of Weigend and co-workers^89−91^ were employed. The frozen core approximation was utilized in all of the post-Kohn–Sham/Hartree–Fock steps. The convergence threshold for the energies was set to 10^–6^*E*_h_, while the default adaptive integration grid of the Mrcc package was used for the XC contributions.

In this study, the exchange and correlation functionals of Perdew, Burke, and Ernzerhof (PBE),^92^ Becke’s 1988 exchange functional (B88),^93^ the correlation functional of Lee, Yang, and Parr (LYP),^94^ and Perdew’s 1986 correlation functional (P86)^95^ were used. The built-in functionals of the Mrcc package were employed in all cases, except for the LC hybrids, where the Libxc library^96,97^ was utilized. In the LC-DH calculations, a locally modified version of Libxc was applied.

The indices Δ**r** and Δ**r**_NTO_ have been implemented in the Mrcc package, while the descriptors ω_CT_ and *d̃*_exc_ were calculated using the TheoDORE^73^ toolbox interfaced with TURBOMOLE v7.1.^98,99^ The MOs for the table of contents image were visualized using the IboView program.^100,101^

The errors utilized for the evaluation of the excitation energies were calculated by subtracting the reference values from the computed ones. The statistical error measures presented in the figures are the mean error (ME), mean absolute error (MAE), standard deviation (SD), maximum absolute error (MAX), and deviation span. All of the computed excitation energies are available in the Supporting Information (SI). In addition, the root-mean-square errors are also included.

### 3.2 Assessed Methods

In this study, the most popular excited-state methods were selected to assess their performance for CT excitations. Of the wave function-based approaches, the CC singles and doubles (CCSD),^102^ second-order algebraic-diagrammatic construction [ADC(2)],^103^ and configuration interaction singles with perturbative second-order correction [CIS(D)]^104^ methods were chosen. The reliable performance of CCSD for Rydberg^105^ and intermolecular CT transitions^62^ has been demonstrated, but some deficiencies for valence^106^ and intramolecular CT^57,107^ excitations have been pointed out. The ADC(2) method is considered to be one of the most efficient fifth-order scaling approaches. It is well-known that its accuracy is similar^56,108,109^ to that of approximate second-order CC (CC2),^110^ but only one system of equations must be solved for each excited state to compute excitation energies and transition moments. The performance of the aforementioned methods is outstanding for valence excitations,^56,106,111^ but their reliability for Rydberg^105^ and challenging CT transitions^62,112^ is often in question. The CIS(D) method is consistently inferior compared with the previous approaches. Nevertheless, it is the simplest method that takes into account electron correlation and double excitations. In addition, the genuine formalism of DH theory for excited states^22^ is also based on it.

For TDDFT calculations, one has a lot of options to select the functionals to be considered. Of course, all of the approaches could not be tested within this study, and the selection had to be made carefully. The failure of the global hybrid functionals for CT transitions because of the wrong long-range behavior of the XC energy has been demonstrated several times.^15,16^ Accordingly, such methods have been completely left out of the comparison. The LC hybrid functionals were originally developed to remedy this shortcoming. One of the first functionals of this class was the CAM-B3LYP approach,^35^ while ωB97X^36^ can be considered as one of the most accurate LC hybrids concerning the benchmark reviews for ground-state properties.^46,47^ The CAMh-B3LYP^113^ and ωB97X-D^114^ methods are also assessed. The former one is similar to CAM-B3LYP, but the adjustable parameters were tuned for CC2 excitation energies, while the latter one is the improved version of ωB97X containing also empirical atom–atom dispersion corrections.

In the case of the original DH functionals, spin-scaling techniques were not applied. One of the most successful of these methods is the empirically parametrized B2GPPLYP approach.^115^ For this functional, two adjustable parameters were tuned for ground-state properties. We note that, of course, such parameters can also be defined using nonempirical considerations through the adiabatic connection formalism. The most noteworthy nonempirical functionals of this kind are the PBE-QIDH^116^ and PBE0-2^117^ methods. The spin-scaling techniques enable higher flexibility of the energy functional and ensure a more accurate description of the chemical properties. One of the most widely used functionals in this category is DSD-PBEP86,^118^ where the XC energy contains four empirical parameters adjusted for ground-state properties. The recently proposed spin-scaled PBE-QIDH methods,^54^ namely, SCS-PBE-QIDH and SOS-PBE-QIDH, feature six and four adjustable parameters, respectively. For these functionals, the spin-scaling parameters were tuned for excited-state calculations, while the remainders were retained from PBE-QIDH. As demonstrated in ref (50), DSD-PBEP86 has excellent accuracy for valence excitations, while its error is significantly higher for Rydberg transitions. Surprisingly, the overall performances of the nonempirical DH functionals are better than that of the empirical B2GPPLYP approach, but their accuracy is not outstanding in comparison with the most recent methods. For intermolecular CT transitions, the B2GPPLYP, DSD-PBEP86, and original PBE-based approaches failed, whereas the spin-scaled variants of PBE-QIDH have not been tested previously for such excitations. The superiority of SCS-PBE-QIDH and SOS-PBE-QIDH in this class was shown in ref (54).

As can be seen, CT transitions could be challenging even for global DH methods. To improve their robustness, several LC analogues were proposed by Goerigk and co-workers. In their first study,^52^ the ωB2GPPLYP functional was introduced, where among the three adjustable parameters, only the range-separation parameter was tuned for excitation energies. Later, the ωPBEPP86 and ωB88PP86 approaches^54^ as well as their SCS and SOS variants were presented. In these cases, all of the adjustable parameters were optimized for the well-known Gordon benchmark set.^74^ On top of this, in the same study, spin-scaled variants for ωB2GPPLYP were also introduced, where the mixing factors, but only they, were tuned for the aforementioned test set. The SCS and SOS variants of such functionals contain seven and five empirical parameters, respectively. LC analogues of nonempirical DHs were also introduced. The RSX-QIDH^48^ and SOS-RSX-QIDH^119^ approaches feature three and four nonempirical parameters, respectively. It should be noted that a similar functional was introduced by Goerigk’s group as well. In their case, the spin-scaling parameters were tuned for excitation energies, while the other parameters were retained from RSX-QIDH. Hereinafter, this functional will be denoted as SOS-RSX-QIDH2.^54^ As shown in refs (29) and (53), the long-range correction significantly improves the Rydberg excitations for ωB2GPPLYP, but the valence results are noticeably inaccurate compared with B2GPPLYP. The ωB2GPPLYP method is superior to RSX-QIDH,^53^ while the empirical spin-scaling optimization significantly improves the results for SOS-RSX-QIDH2.^54^ The SCS-ωPBEPP86 and SOS-ωPBEPP86 functionals can be considered as the most recommended approaches from Goerigk’s group. However, as was pointed out in ref (51), SOS-ωPBEPP86 is not consistently better than either the PBE0-2 or DSD-PBEP86 functional. The ωB2GPPLYP approach could be an appropriate choice to describe intermolecular CT excitations, while the more highly parametrized SOS-ωPBEPP86 failed for such transitions.^51^ In addition, as we will see, the long-range correction has a less significant influence on the intramolecular CT excitations, in contrast to what the authors of ref (54) claimed.

In the case of the more elaborate RS-DH functionals, both the exchange and correlation contributions are range-separated.^41^ A genuine spin-scaled excited-state analogue was recently proposed in our study.^50^ Later, an ADC(2)-based ansatz^120^ was also introduced.^51^ The SOS variants, namely, RS-PBE-P86/SOS-CIS(D) and RS-PBE-P86/SOS-ADC(2), contain only three adjustable parameters, while the SCS analogues have four empirical parameters. All of the factors were tuned for excited-state calculations. We note that the same parameter set was optimal for both the CIS(D)- and ADC(2)-based functionals. As shown in the original papers, the CIS(D)-based functionals can be considered as the most robust alternatives within the genuine DH theory. Their accuracy is similar to that of the best performers, and in addition, appropriate results can be achieved for the most challenging CT transitions. On top of this, the ADC(2)-based ansatz significantly improves the results for valence excitations and also enables us to evaluate the transition moments at a higher level. It was demonstrated that the suggested ADC(2)-based functionals provide the most robust and accurate excitation energies within the DH theory by far, while the relative error of the oscillator strengths is reduced by 65% compared to the best genuine DH functionals. To help the reader, the attributes of all of the functionals discussed in this subsection are collected in Table 1, while the mixing factors of different types of contributions and the range-separation parameters for the functionals are collected in the SI.

**Table 1:** Functionals Assessed in the Benchmark Calculations

functional	exchange	correlation	class	spin scaling	number of parameters	ref
RS-PBE-P86/SCS-ADC(2)	PBE	P86	RS-DH	yes	4	(51)
RS-PBE-P86/SOS-ADC(2)	PBE	P86	RS-DH	yes	3	(51)
RS-PBE-P86/SCS-CIS(D)	PBE	P86	RS-DH	yes	4	(50)
RS-PBE-P86/SOS-CIS(D)	PBE	P86	RS-DH	yes	3	(50)
SCS-ωPBEPP86	PBE	P86	LC-DH	yes	7	(54)
SOS-ωPBEPP86	PBE	P86	LC-DH	yes	5	(54)
SCS-ωB88PP86	B88	P86	LC-DH	yes	7	(54)
SOS-ωB88PP86	B88	P86	LC-DH	yes	5	(54)
SCS-ωB2GPPLYP	B88	LYP	LC-DH	yes	7	(54)
SOS-ωB2GPPLYP	B88	LYP	LC-DH	yes	5	(54)
SOS-RSX-QIDH	PBE	PBE	LC-DH	yes	4	(119)
SOS-RSX-QIDH2	PBE	PBE	LC-DH	yes	5	(54)
RSX-QIDH	PBE	PBE	LC-DH	no	3	(48)
ωB2GPPLYP	B88	LYP	LC DH	no	3	(52)
SCS-PBE-QIDH	PBE	PBE	global DH	yes	6	(54)
SOS-PBE-QIDH	PBE	PBE	global DH	yes	4	(54)
DSD-PBEP86	PBE	P86	global DH	yes	4	(118)
PBE0-2	PBE	PBE	global DH	no	2	(117)
PBE-QIDH	PBE	PBE	global DH	no	2	(116)
B2GPPLYP	B88	LYP	global DH	no	2	(115)
CAM-B3LYP	B88	LYP	LC hybrid	N/A	3	(35)
CAMh-B3LYP	B88	LYP	LC hybrid	N/A	3	(113)
ωB97X	B97	B97	LC hybrid	N/A	17	(36)
ωB97X-D	B97	B97	LC hybrid	N/A	18	(114)

### 3.3 Benchmark Sets

To assess the performance of the approaches, three different benchmark sets were selected from the literature. As was previously emphasized, these compilations can currently be considered as the most comprehensive ones for CT excitations. First, the test set of Loos, Jacquemin, and co-workers^57^ is analyzed. This compilation, which is hereafter called the LJCT set, contains 30 intramolecular (π → π* and *n* → π*) CT transitions for 17 π-conjugated compounds: aminobenzonitrile, aniline, azulene, benzonitrile, benzothiadiazole, dimethylaminobenzonitrile, dimethylaniline, dipeptide, β-dipeptide, hydrogen chloride, nitroaniline, nitrobenzene, nitrodimethylaniline, nitropyridine *N*-oxide, *N*-phenylpyrrole, phthalazine, and quinoxaline. The ground-state geometries, except for the dipeptide molecules, were obtained at the CCSD(T)^121^ or CC3^64^ level using the cc-pVTZ basis sets. For this benchmark set, the high-quality CC-based^63,64^ theoretical best estimates (TBEs) with cc-pVTZ basis sets were suggested as the reference using an incremental approach as
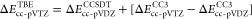
5

The comprehensive intermolecular CT benchmark set proposed by Szalay et al.^62^ contains 14 excitation energies for nine molecular complexes: ammonia–fluorine (NH_3_–F_2_), acetone–fluorine, pyrazine–fluorine, ammonia–oxygen difluoride, acetone–nitromethane, ammonia–pyrazine, two different pyrrole–pyrazine structures, and tetrafluoroethylene–ethylene. With one exception, the equilibrium structures were obtained by full-dimensional ground-state optimizations using the CC2 method with the cc-pVDZ basis sets. The resulting geometries ensure, for most of the transitions, almost perfect charge separation between the fragments. Additional calculations were carried out for the NH_3_–F_2_ system, in which the distance between the fragments was increased with respect to the equilibrium structure and the accuracy of the approaches was inspected as a function of the separation.^112^ The reference energies were calculated at the CCSDT-3 level^65^ using the cc-pVDZ basis sets in all cases.

Finally, four aryl–tetracyanoethylene (Ar–TCNE) complexes (Ar = benzene, toluene, *o*-xylene, and naphthalene) proposed by Baer and co-workers^67^ are assessed. This test set, like to the previous one, contains only intermolecular CT transitions. For the obtained ground-state structures, the equilibrium interplanar distances were between 3.6 and 3.9 Å using the B3LYP functional^122^ with the cc-pVDZ basis set. As the reference values are experimental gas-phase results in the original paper, the first bright excited state for each approach is compared to them. The same analysis was carried out in some of the most recent LC-DH studies.^27,28,123^

## 4 Results

### 4.1 Intramolecular Excitations

**Figure 1:**
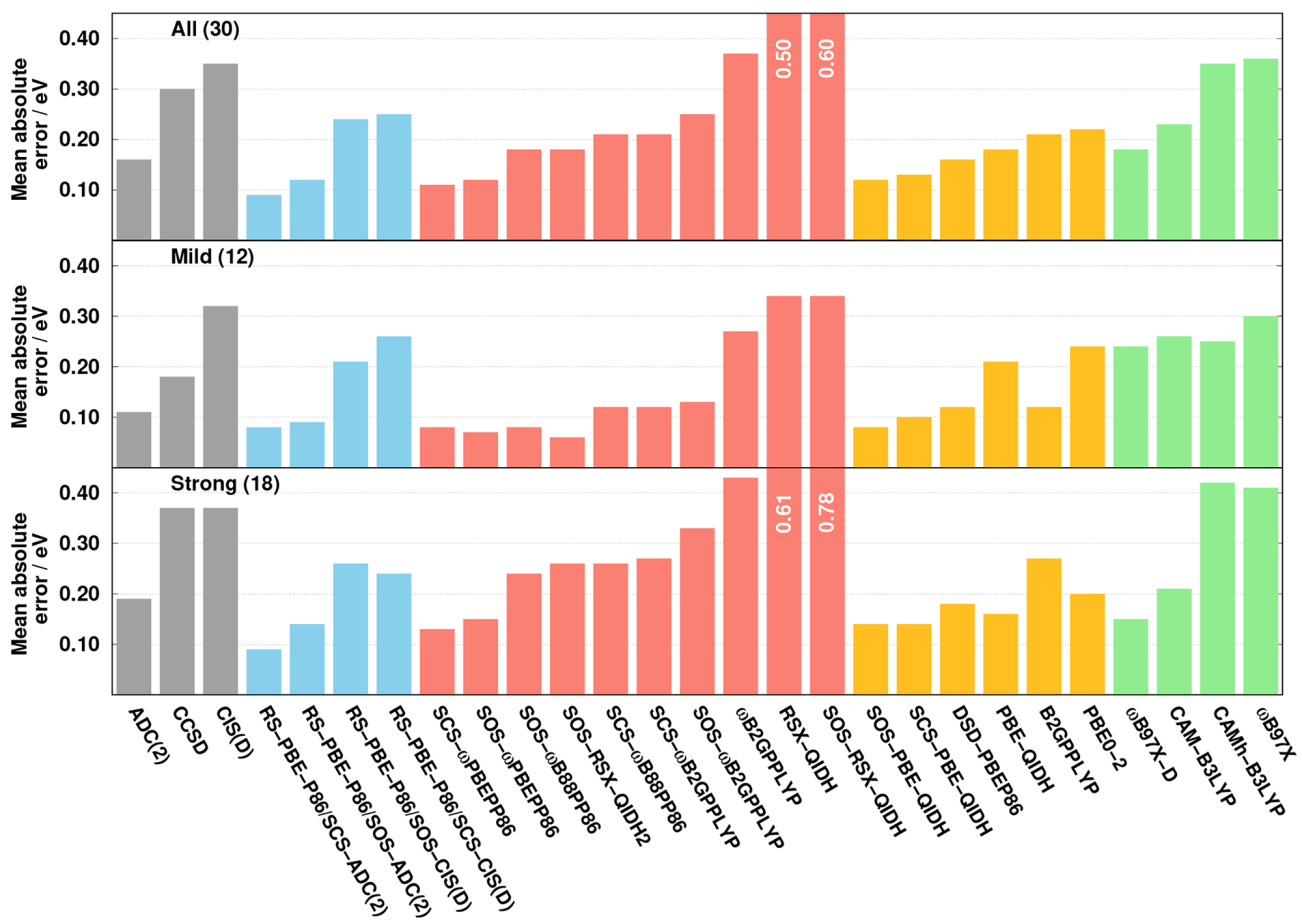
MAEs for the LJCT test set^57^ for different types of CT transitions using the cc-pVTZ basis set with the corresponding auxiliary bases. The numbers of transitions are given in parentheses. The wave function, RS-DH, LC-DH, DH, and LC hybrid methods are presented in gray, blue, red, orange, and green, respectively. The CCSD values were taken from ref (57)

First, the LJCT test set^57^ is discussed in detail. In their original paper, the authors split up the excitations into mild and strong CT transitions. The selection was based on the distance of the electron–hole separation analyzing the ADC(2) wave function. This characterization is kept unchanged in the first part of our study. Accordingly, the results for different types of transitions are presented in Figure 1. Inspecting the bars, we can observe that the best overall performance is attained by RS-PBE-P86/SCS-ADC(2), with a MAE of 0.09 eV. It is higher by 0.02 eV for SCS-ωPBEPP86, while the next methods are the RS-DH RS-PBE-P86/SOS-ADC(2), the global DH SOS-PBE-QIDH, and the LC-DH SOS-ωPBEPP86 approaches. In these cases, the MAE is uniformly 0.12 eV, which is still outstanding. Interestingly, three different classes of methods can be identified among the best performers. This means that outstanding results can be achieved even without long-range correction as well. A somewhat higher error is obtained for SCS-PBE-QIDH, while the performance of DSD-PBEP86 is similar to that of ADC(2). The MAE is still below 0.20 eV for the PBE-QIDH, SOS-ωB88PP86, SOS-RSX-QIDH2, and ωB97X-D approaches. On the basis of these results, we can conclude that the empirical tuning of the excitation energies in the case of SOS-RSX-QIDH2 is highly favorable, as the nonempirical counterpart is inferior with a MAE of 0.60 eV. In addition, for this benchmark set, ωB97X-D can be considered as the most reliable LC hybrid. A similar finding was obtained in ref (57). Surprisingly, in the case of B2GPPLYP, where the MAE is 0.21 eV, the long-range correction significantly increases the error, as it is 0.37 eV for ωB2GPPLYP. The performance of SCS-ωB2PPLYP and SCS-ωB88PP86 is similar to that of B2GPPLYP. In other words, the higher level of parametrization for SCS-ωB2PPLYP fixed the shortcomings of ωB2PPLYP. The PBE-QIDH approach is a bit better than PBE0-2, while the CAM-B3LYP, SOS-ωB2GPPLYP, and genuine spin-scaled RS-PBE-P86 functionals, with MAEs of around 0.25 eV, are still more accurate than CCSD. Interestingly, the excited-state-tuned CAMh-B3LYP is less reliable than CAM-B3LYP. The RSX-QIDH approach is not recommended at all, as the error is 0.50 eV in this case.

Interesting observations can be made if the different types of CT transitions are inspected separately. The methods with the best overall performance, namely, the spin-scaled variants of the RS-PBE-P86/ADC(2), PBE-QIDH, and ωPBEPP86 functionals, are also the most suitable ones for the mild CT excitations. Interestingly, the SOS-RSX-QIDH2 and SOS-ωB88PP86 methods are outstanding as well. In all of the cases, the MAE is below 0.10 eV. The DSD-PBEP86 and SOS/SCS-ωB2GPPLYP approaches are as accurate as ADC(2). The B2GPPLYP functional is more reliable than CCSD, as the MAEs are 0.12 and 0.18 eV, respectively. It exceeds 0.20 eV for the rest of the methods. This order, apart from a few exceptions, is highly similar to the overall performance, but the picture somewhat changes when the strong CT excitations are inspected. The MAE is below 0.10 eV only for RS-PBE-P86/SCS-ADC(2), while it is higher by 0.04 eV for SCS-ωPBEPP86. The RS-PBE-P86/SOS-ADC(2) and SOS/SCS-PBE-QIDH approaches are outstanding in this regard as well. Again, even for the strong CT transitions, some of the best performers do not contain long-range correction. The next two functionals are the ωB97X-D and SOS-ωPBEPP86 methods, with a MAE of 0.15 eV, and PBE-QIDH and DSD-PBEP86 are also better than the remaining LC-DH functionals. Surprisingly, the LC-DH (SOS-)RSX-QIDH and ωB2GPPLYP approaches are inferior, while ωB97X and CAMh-B3LYP are also not recommended for such excitations. This is also true for the SCS/SOS-ωB2GPPLYP and SCS/SOS-ωB88PP86 functionals. The error is more moderate compared with ωB2GPPLYP but still significantly higher in comparison with the best performers.

An additional important measure can be the balance of the errors for the mild and strong CT transitions. To quantify that, we subtracted these two MAEs for each functional. On the basis of our results, the most robust performances are attained by the SCS-ADC(2) and SCS-CIS(D) variants of RS-PBE-P86, with the differences of 0.01 and 0.02 eV, respectively. The results are also well-balanced for the PBE-based global approaches, with a difference of around 0.05 eV, while the same values were obtained for the SOS-ADC(2) and SOS-CIS(D) variants of RS-PBE-P86 as well as for CAM-B3LYP and SCS-ωPBEPP86. For all of the other LC-DH functionals, this difference is significantly higher. Surprisingly, except for ωB97X-D, the overall errors obtained for strong CT transitions are notably higher for all of the LC-DH and LC hybrid functionals compared with the mild transitions. This effect is more moderate even for global DHs.

**Figure 2:**
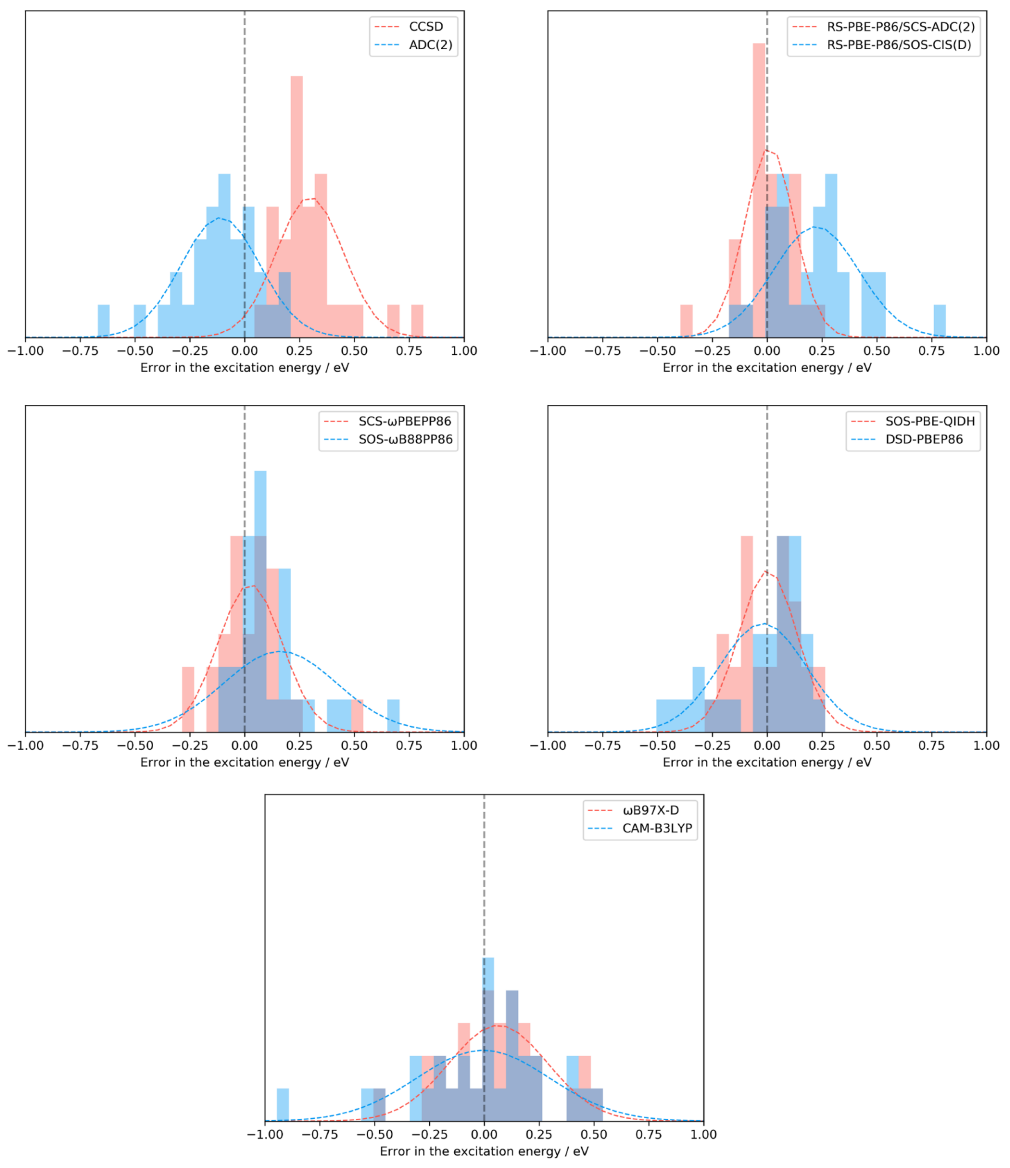
Error patterns for the LJCT test set^57^ for the representative methods of the various categories.

In the following, further analysis is carried out for a few selected approaches. For each class of methods, the two best performers are taken. For the RS-DH, LC-DH, and global DH functionals, these two approaches differ only in the spin-scaling techniques. Consequently, to ensure a comprehensive comparison, the third best performers are selected in these cases instead of the second ones. The error patterns are visualized in Figure 2. As can be seen, an almost perfect ME is achieved by the RS-PBE-P86/SCS-ADC(2), SOS-PBE-QIDH, and CAM-B3LYP functionals, although for the last one the SD is significantly higher. The ME is 0.03 eV for SCS-ωPBEPP86. Interestingly, for ωB97X-D, SOS-ωB88PP86, and RS-PBE-P86/SOS-CIS(D), the excitation energies are also overestimated on average by 0.06, 0.16, and 0.22 eV, respectively, while they are slightly underestimated for DSD-PBEP86. The largest range is covered by the wave function-based methods, as the errors are −0.11 and 0.30 eV for the ADC(2) and CCSD approaches, respectively. For the SDs, again RS-PBE-P86/SCS-ADC(2) is superior, with an error of 0.11 eV. For the SOS-PBE-QIDH and SCS-ωPBEPP86 functionals, it does not exceed 0.15 eV, whereas the deviation is somewhat larger for the wave function-based methods. In this regard, the performances of the RS-PBE-P86/SOS-CIS(D) and DSD-PBEP86 approaches are identical, with an SD of 0.20 eV, while it is 0.22 eV for the SOS-ωB88PP86 functional.

The lowest MAX, precisely 0.24 eV, is attained by SOS-PBE-QIDH. It also demonstrates that highly reliable results can be obtained for intramolecular CT excitations without range-separation techniques. This measure is also outstanding for RS-PBE-P86/SCS-ADC(2) with a value of 0.34 eV, while the MAX is still below 0.50 eV for DSD-PBEP86 and SCS-ωPBEPP86. Resulting in the same order, the lowest error spans are also achieved by these approaches, with errors of 0.47, 0.58, 0.71, and 0.77 eV, respectively. The MAX is 0.51 eV for ωB97X-D, which is still acceptable, but the error span of 0.97 eV is a bit unfavorable. In contrast, for the former measure, a somewhat less satisfactory result is obtained for RS-PBE-P86/SOS-CIS(D), while the error span is 0.90 eV. In the case of the wave function-based methods, the MAX is more moderate for ADC(2), while the other measure is more acceptable for CCSD. Nevertheless, both methods are far from the best functionals. For both measures, the CAM-B3LYP and SOS-ωB88PP86 approaches are inferior, as the MAX (error span) values are 0.91 (1.25) and 1.12 (1.35) eV, respectively.

**Figure 3:**
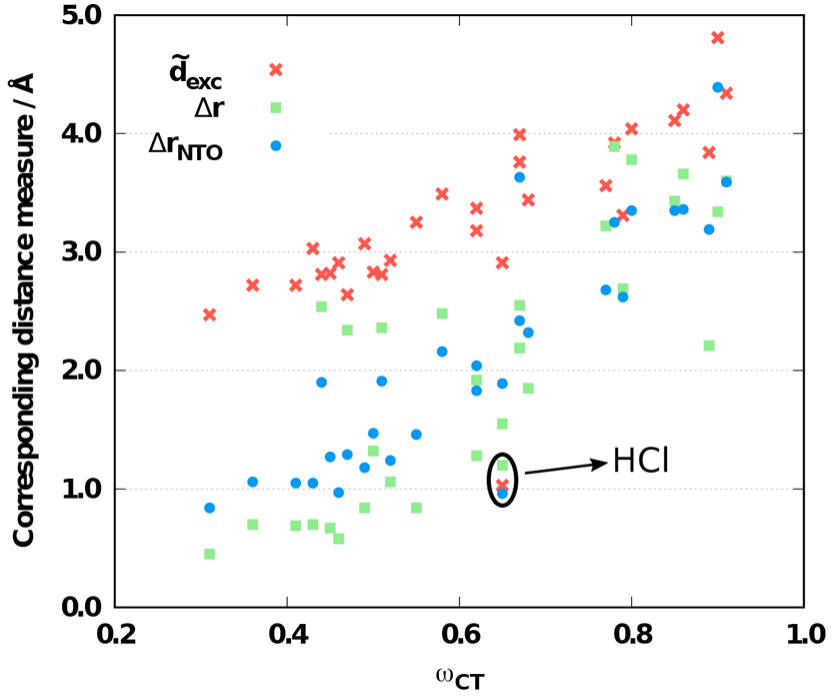
Correlation between ω_CT_ and distance measures for the LJCT test set.^57^

The errors can also be inspected as a function of the CT metrics. First, we discuss the correlation between the descriptors. For this purpose, the Δ**r** and Δ**r**_NTO_ indices were calculated using the singles part of the ADC(2) wave function. The NTOs required for the latter measure were obtained in the same way. To the best of our knowledge, the TheoDORE program package also takes into account only single excitations from the ADC(2) wave function to calculate the ω_CT_ and *d̃*_exc_ descriptors. With this procedure, the descriptors were obtained at the same level, and the uncertain quality of the TDDFT methods can be excluded. The fragmentation of the molecules used to calculate the ω_CT_ index is available in the SI. The results are collected in Figure 3. As can be seen, the correlation between the corresponding hole–particle distance measure and the total amount of charge separation is very clear. That is, especially for the *d̃*_exc_ and Δ*r*_NTO_ descriptors, the hole–particle distances become more significant with increasing ω_CT_. This trend is broken by the HCl diatomic molecule, where a notable ω_CT_ value can be observed but the hole–particle distance is not significant. For the Δ**r** index, the pattern is somewhat less consistent, as several decreasing distance values can be found with increasing ω_CT_, especially in the range of 0.5 to 0.7. In contrast, the NTO-based analogue follows the trend properly. For small ω_CT_, the obtained values are a bit higher compared with Δ**r**, while it starts to increase consistently from 1.0 Å with increasing ω_CT_. The behavior of the *d̃*_exc_ descriptor is similarly regular, but noticeably higher values were attained for small charge separation in comparison with Δ**r**_NTO_. This difference decreases rapidly with increasing ω_CT_. The highest values, around 4.5 Å, were obtained for the β-dipeptide molecule. On the basis of these numerical experiences, we can conclude that at least for the LJCT test set, the Δ**r**_NTO_, *d̃*_exc_, and ω_CT_ descriptors can be arbitrarily used to assess the correlation between the error in the excitation energies and the extent of the charge separations without any bias. For convenience, hereinafter we use the Δ**r**_NTO_ index, as it has been implemented in our program system.

**Figure 4:**
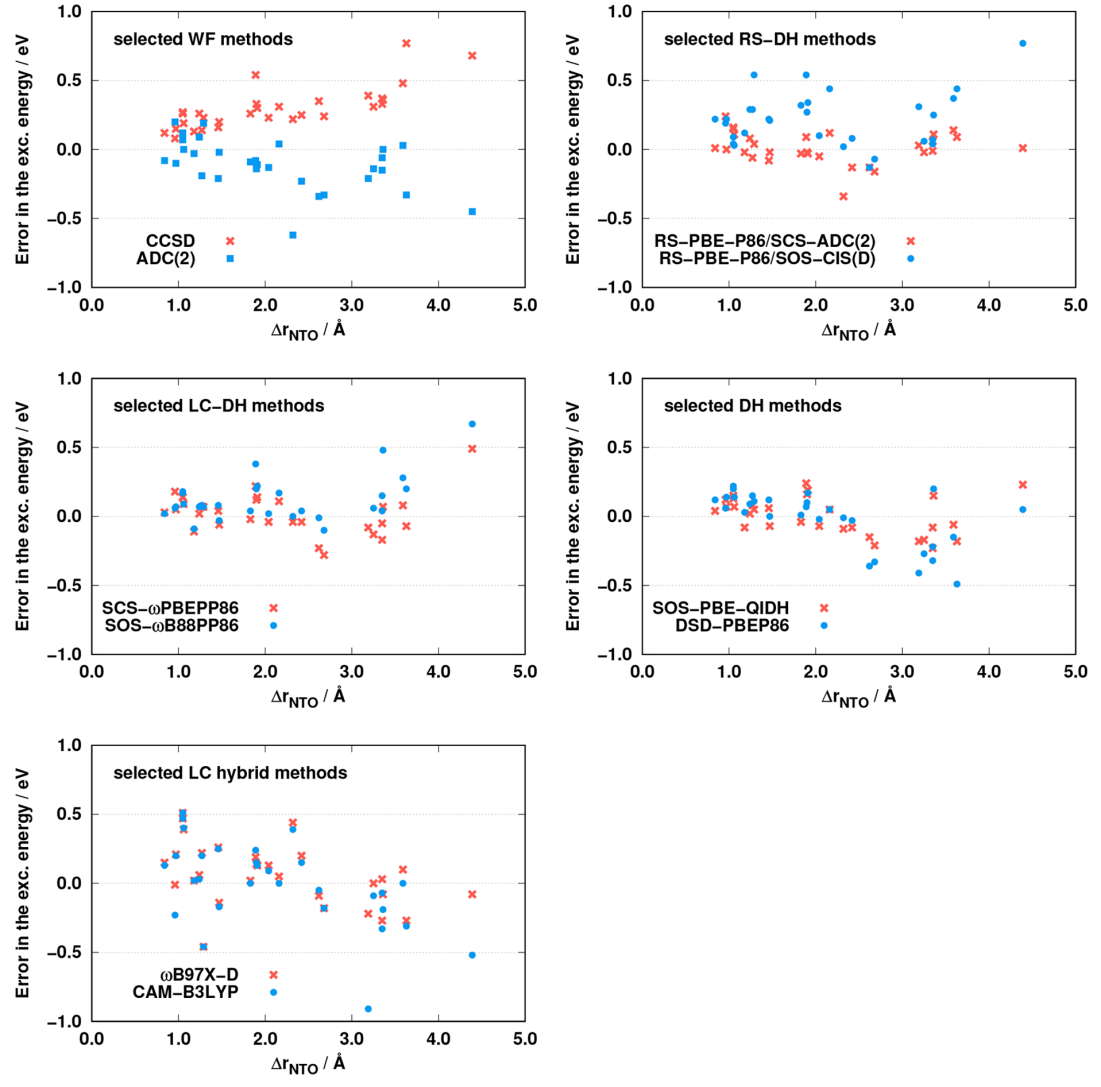
Errors of the corresponding excitation energies as functions of the Δ**r**_NTO_ measure for the LJCT test set.^57^

It is desirable to determine whether there is a connection between the inaccuracy of the methods and the hole–particle distance for such transitions. We note that of course the accuracy is affected by many circumstances. Nevertheless, as we will see, clear trends can be determined for some of the approaches. For the selected methods, the errors as functions of the CT descriptor are plotted in Figure 4. Inspecting the results for the wave function-based methods, we can conclude that a strong correlation is present for the CCSD approach, as the error is monotonically blue-shifted with increasing distance. This outcome is fairly unfavorable, as the excitation energies are overestimated for small distances as well. In the case of ADC(2), the correlation is more moderate, and the error is somewhat red-shifted. For the RS-DH and LC-DH approaches, a correlation cannot be recognized. In the case of RS-PBE-P86/SCS-ADC(2) and SCS-ωPBEPP86, as was previously discussed, the error fluctuates within a narrow range, while it is a bit more hectic for the RS-PBE-P86/SOS-CIS(D) and SOS-ωB88PP86 approaches. The salient error of the CIS(D)-based approaches at large distances belongs to the β-dipeptide molecule including a large fraction of double excitations. A clear but mild correlation can be determined for the global DH methods. As can be seen, this effect is somewhat more intense for DSD-PBEP86 compared with SOS-PBE-QIDH. Not surprisingly, the error is red-shifted in both cases with increasing Δ**r**_NTO_ index. The excitation energies are overestimated at small distances, especially for DSD-PBEP86, whereas they are consistently underestimated for Δ**r**_NTO_ > 2.5 Å. For the LC hybrids, the errors are strongly affected by the charge separation. A systematic red shift can be observed with increasing distance, as the excitation energies are mainly overestimated for small distances whereas the ME is negative for transitions with Δ**r**_NTO_ > 3.0 Å.

### 4.2 Intermolecular Excitations

**Figure 5:**
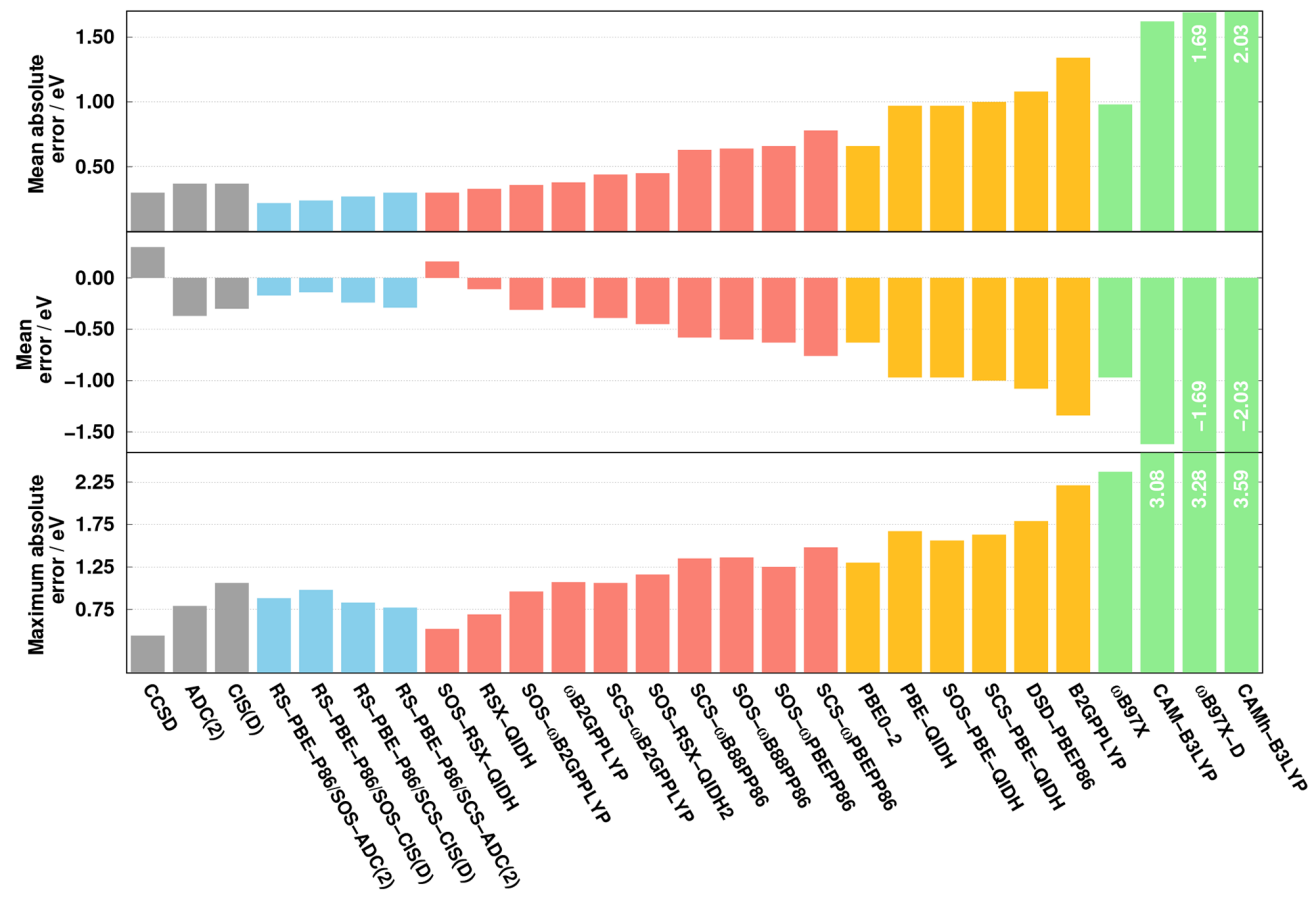
Error measures for the calculated excitation energies for the test set of Szalay^62^ using the cc-pVDZ basis set with the corresponding auxiliary bases. The wave function, RS-DH, LC-DH, DH, and LC hybrid methods are presented in gray, blue, red, orange, and green, respectively. The CCSD values were taken from ref (62).

Next, we assess intermolecular CT excitations using the benchmark set of Szalay and co-workers.^62^ For most of the transitions, an almost perfect charge separation between the fragments of the complexes is ensured. The error measures are visualized in Figure 5. Inspection of the overall performances shows that the best results are attained by the RS-DH approaches. The lowest MAEs are 0.22 and 0.24 eV for the SOS-ADC(2) and SOS-CIS(D) variants of RS-PBE-P86, respectively, while the error is higher by 0.05 eV for the SCS analogues. Similar performance is observed for SOS-RSX-QIDH and CCSD, with a MAE of 0.30 eV. The error is still acceptable for RSX-QIDH and ωB2GPPLYP as well as for the SOS variant of the latter. These methods are practically as accurate as the fifth-order scaling wave function-based methods, with MAEs of around 0.37 eV. Interestingly, these LC-DH functionals were inferior for intramolecular CT transitions. Significantly higher errors were obtained for the rest of the functionals, with MAEs around 0.45 eV for the SCS-ωB2GPPLYP and SOS-RSX-QIDH2 approaches. Similar performances were attained by SCS/SOS-ωB88PP86 and SOS-ωPBEPP86 compared to the best global DH, namely, PBE0-2. The error is around 0.65 eV in these cases. Despite the excellent performance for intramolecular excitations, SCS-ωPBEPP86 is inferior in the LC-DH class. The original PBE-QIDH approach is somewhat more reliable than its SCS and SOS analogues, but the error is 1.00 eV for these functionals. Similar accuracy is observed for ω-B97X, which can be considered as the best LC hybrid. The DSD-PBEP86 and B2GPPLYP approaches are the two inferior methods in the global DH class, with MAEs of 1.08 and 1.34 eV, respectively. The CAM(h)-B3LYP and ωB97X-D functionals are completely inadequate choices to describe such excitations, as the errors are higher than 1.60 eV. Interestingly, similar to the case of intramolecular transitions, the excited-state-tuned CAMh-B3LYP functional is less accurate than CAM-B3LYP.

As the excitation energies are systematically underestimated, especially for the inferior methods, a highly similar order can be observed for the MEs. The lowest errors are attained by SOS-RSX-QIDH and RS-PBE-P86/SOS-CIS(D), with the MEs of −0.11 and −0.14 eV, respectively. It is still acceptable for SOS-RSX-QIDH and RS-PBE-P86/SOS-ADC(2), while the ME is significantly more favorable compared with the MAE for ωB2GPPLYP and its spin-scaled variants. Accordingly, in this regard (SOS-)ωB2GPPLYP is as accurate as RS-PBE-P86/SCS-ADC(2), while the SCS-ωB2GPPLYP approach gets closer to the CIS(D) and ADC(2) methods. For the rest of the functionals, the order does not change, and the ME is practically equal to the MAE with the corresponding minus sign. The excitation energies are overestimated for CCSD and RSX-QIDH, whereas they are underestimated for the other functionals.

The lowest MAX, precisely 0.44 eV, is achieved by CCSD. The SOS-RSX-QIDH and RSX-QIDH approaches have outstanding performances as well, with maximum errors of 0.52 and 0.69 eV, respectively. The next functionals are the SCS-ADC(2) and SCS-CIS(D) variants of RS-PBE-P86. They are as reliable as ADC(2), with a MAX of around 0.80 eV. It is still below 1.00 eV for their SCS variants and for the SOS-ωB2GPPLYP approach. A few LC-DH methods can be found with MAX values between 1.05 and 1.25 eV, while the maximum error is 1.30 eV in the case of the best global DH, namely, PBE0-2. Practically similar performance is attained by the SCS and SOS variants of ωB88PP86, with a MAX of around 1.35 eV, while the highest error of the LC-DH class, 1.48 eV, is obtained for SCS-ωPBEPP86. The MAX exceeds 1.50 eV for PBE-QIDH and its spin-scaled variants, and again, the DSD-PBEP86 and B2GPPLYP approaches are inferior in this class, with MAX values of 1.79 and 2.21 eV, respectively. Interestingly, even higher maximum errors can be obtained for the LC hybrid functionals. In these cases, the lowest MAX is 2.37 eV, and it can exceed 3.50 eV.

**Figure 6:**
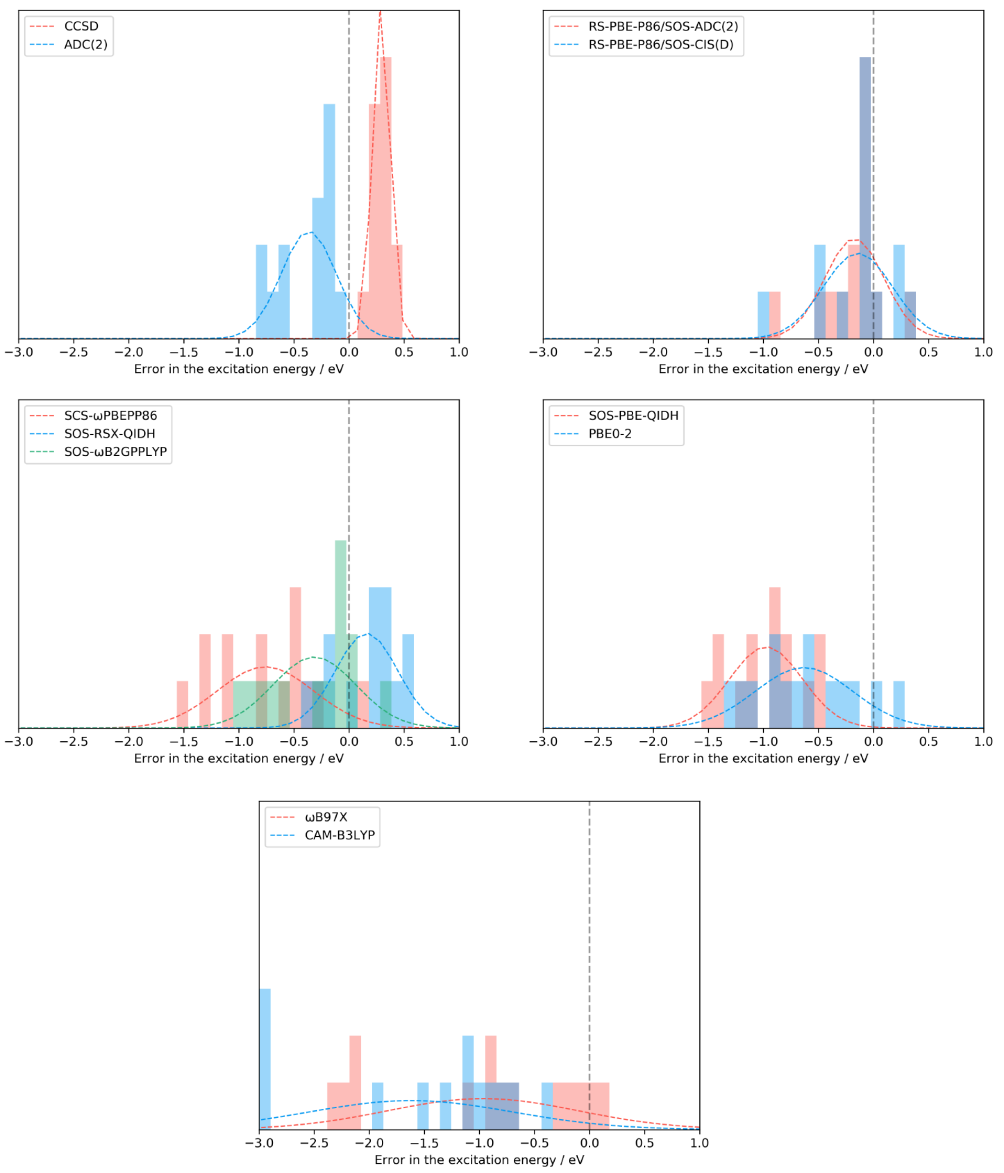
Error patterns for the test set of Szalay^62^ for representative methods of the various categories.

Again, some of the best performers are appointed for further analysis. The selection was carried out using similar considerations as for the intramolecular transitions. In addition, the LC-DH class was supplemented with the SCS-ωPBEPP86 functional since neither of the two best performers of this class was discussed in the previous detailed analysis. In this part of the study, the SDs and error spans are assessed for the selected methods. For this purpose, the results are visualized in Figure 6. As can be seen, the lowest SD by far, 0.08 eV, is attained for CCSD. The next approach is the ADC(2) method, with a deviation of 0.26 eV, while it is higher by 0.02 eV for RS-PBE-P86/SOS-ADC(2). The SD does not exceed 0.30 eV for the best LC-DH, namely, SOS-RSX-QIDH, while it is still acceptable for the RS-PBE-P86/SOS-CIS(D) and SOS-PBE-QIDH approaches. In these cases, the deviation is below 0.35 eV, whereas it is 0.39, 0.46, and 0.46 eV for the SOS-ωB2GPPLYP, PBE0-2, and SCS-ωPBEPP86 functionals, respectively. Again, the LC hybrids are inferior because the SD is at least 0.90 eV for these functionals. The lowest error spans are also attained by the wave function-based methods, which are 0.29 and 0.70 eV for the CCSD and ADC(2) approaches, respectively. The best functional is SOS-RSX-QIDH, where the span does not exceed 1.00 eV. Surprisingly, the next-best performer is the SOS-PBE-QIDH approach, with an error of 1.11 eV, while this measure is 1.18 eV for RS-PBE-P86/SOS-ADC(2). The error span is still below 1.30 eV for RS-PBE-P86/SOS-CIS(D) and SOS-ωB2GPPLYP, whereas significantly higher values were obtained for PBE0-2 and SCS-ωPBEPP86. In these cases, the span is 1.52 and 1.62 eV, respectively, while it is around 2.50 eV for the LC hybrid functionals.

**Figure 7:**
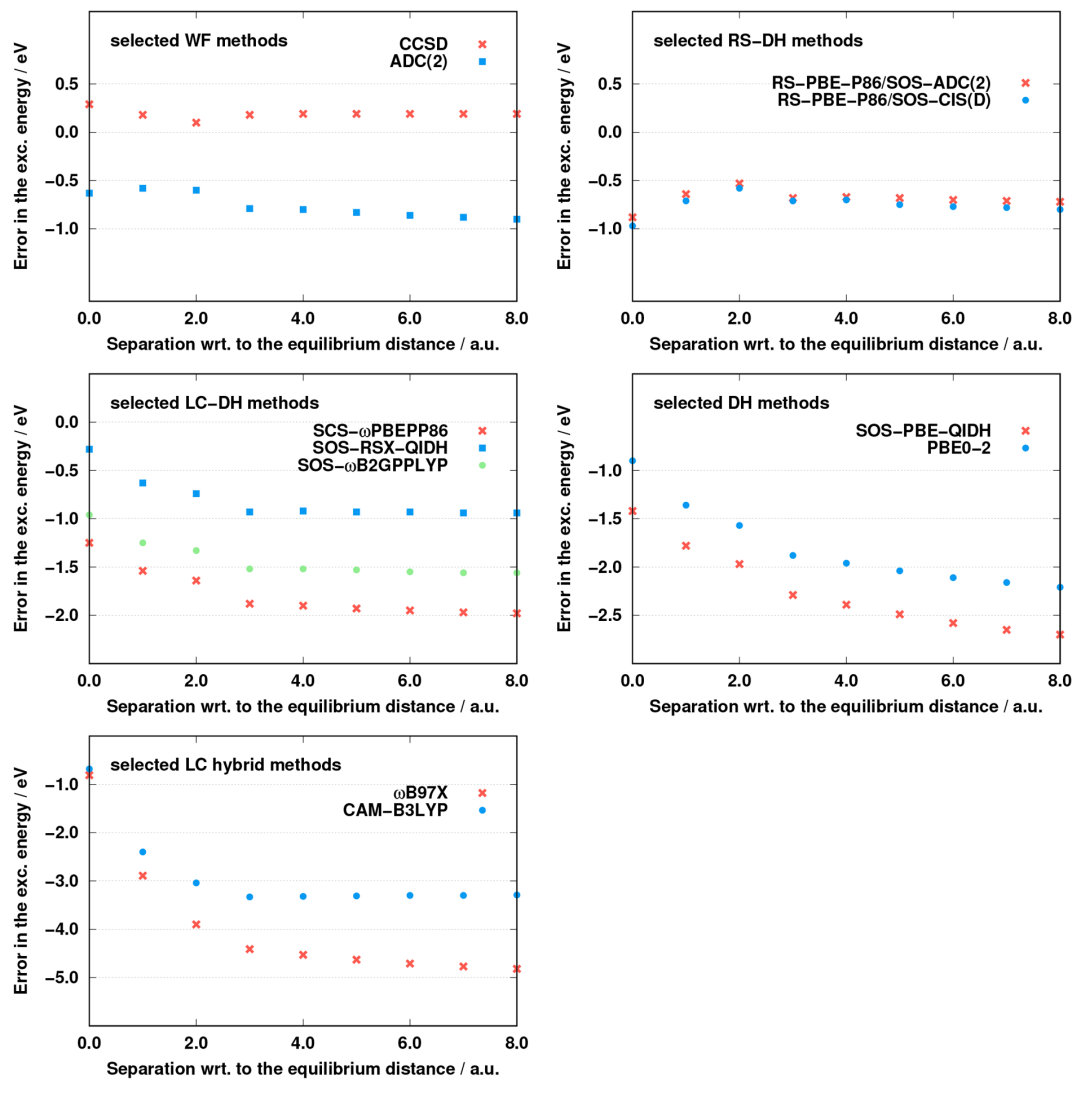
Error of the corresponding excitation energy as a function of the separation with respect to the equilibrium distance of the NH_3_–F_2_ system. It should be noted that all of the vertical axes span 3.0 eV, except for the LC hybrids, where the range is twice as large.

Next, the correct behavior of the XC energy is tested. For this purpose, the separation between the fragments of the NH_3_–F_2_ complex is increased with respect to the equilibrium structure, and the accuracy of the selected methods is inspected at different intermolecular distances. The initial intermolecular distance is around 4.67 au. A similar analysis was carried out for higher-order CC methods in ref (112). The results are presented in Figure 7. As can be seen, the accuracy is hardly affected by the distance for the wave function-based methods. In the case of CCSD, the excitation energy is overestimated in the entire range. For small separation, the error is red-shifted with increasing distance, while it starts to increase very slowly from a separation of 2.0 au. The opposite findings can be seen for ADC(2). In this case, the excitation energy is underestimated. The error starts to decrease for small separation as well, but the slope has a different sign compared with CCSD. Reaching the same point, the error starts to increase, but the growth is somewhat more significant for ADC(2). Very promising results were obtained for the RS-DH approaches. In these cases, the curves have a fairly similar shape, as was discussed for ADC(2). The slope is a bit steeper in the first region, but the drop from the 2.0 au separation is more moderate compared with that for ADC(2). The errors fluctuate in a very small range within the entire region inspected. Significant differences between the genuine and ADC(2)-based ansatze cannot be observed.

The results are significantly affected by the separation in the case of the LC-DH functionals. For such methods, the excitation energies are, not surprisingly, underestimated. In the first region, the errors are highly red-shifted with increasing separation, and the slope is fairly steep. The error ranges are higher compared with the RS-DH functionals, while the plateau is also reached at a higher separation. The best performance in this regard is attained by SOS-ωB2GPPLYP, with an error range of 0.70 eV, while it is 1.15 eV for SCS-ωPBEPP86 and SOS-RSX-QIDH. The error curve flattens at a separation of 3.0 au. The effect of the distance is even more drastic for the global DH approaches. In these cases, the errors increase rapidly by 1.00 eV up to a separation of 3.0 au, while the constant error cannot be reached within the range inspected. The PBE0-2 functional has a bit better performance compared with SOS-PBE-QIDH, but the limitation of both functionals is demonstrated. The LC hybrid approaches fail completely for this test. The curves have a similar shape as observed for the LC-DH methods, but the shortcomings are even more significant. For the CAM-B3LYP method, the error increases by 2.00 eV in the first few steps, while it is 3.00 eV for ωB97X. For the former, the plateau is reached at 3.0 au, while for the latter, the slow decline does not stop even for higher separations.

In addition to the shape of the curves, it is also important to quantify the absolute errors at complete separation. For this purpose, the errors were calculated at a separation of 100 au as well. In this regard, not surprisingly, the best performance is attained by CCSD, with an error of 0.20 eV. The difference is 0.85 and 0.95 eV for the SOS-ADC(2) and SOS-CIS(D) variants of RS-PBE-P86, respectively, while it does not exceed 1.00 eV for SOS-RSX-QIDH. The ADC(2) approach is also acceptable, with an error of 1.10 eV. For the remainders, the inaccuracy is significantly larger. The errors are 1.65 and 2.26 eV for the SOS-ωB2GPPLYP and SCS-ωPBEPP86 functionals, respectively. A somewhat worse result is obtained for PBE0-2, while the error already exceeds 3.00 eV for ωB97X. Furthermore, the excitation energy is underestimated by 3.32 and 5.41 eV for the SOS-PBE-QIDH and CAM-B3LYP approaches, respectively.

**Figure 8:**
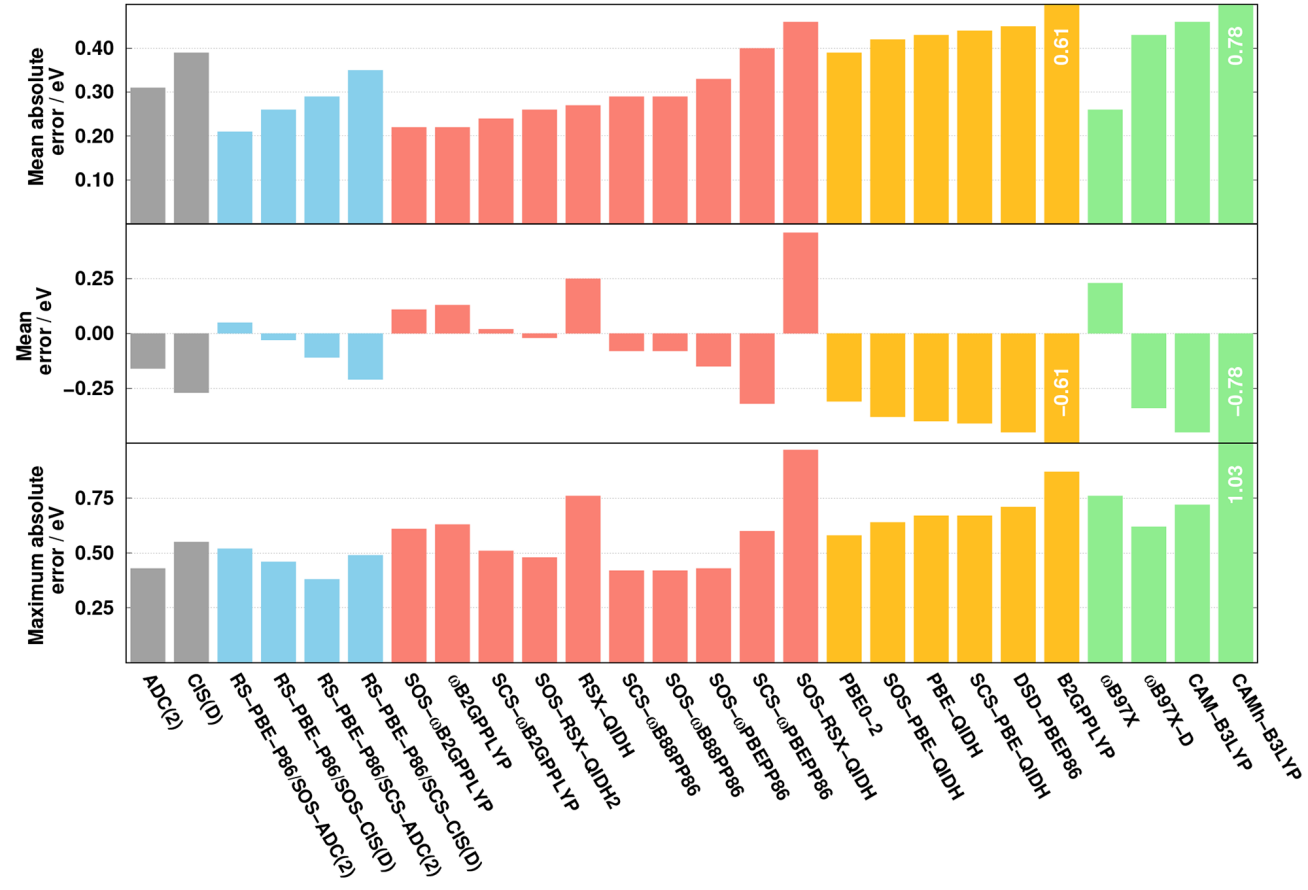
Error measures for the calculated singlet excitation energies for the Ar–TCNE test set^67^ using the cc-pVTZ basis set with the corresponding auxiliary bases. The wave function, RS-DH, LC-DH, DH, and LC hybrid methods are presented in gray, blue, red, orange, and green, respectively.

Finally, the Ar–TCNE benchmark set of Baer and co-workers^67^ is assessed. As the compilation contains only four intermolecular CT excitations and the reference values are experimental results, the outcomes are only briefly discussed, focusing on the best performers of each class. The results are visualized in Figure 8. As can be seen, the lowest MAE, precisely 0.21 eV, is attained by RS-PBE-P86/SOS-ADC(2). Outstanding results are achieved by ωB2GPPLYP and its spin-scaled variants as well. The MAE is still below 0.25 and 0.30 eV for the SOS-CIS(D) and SCS-ADC(2) analogues of RS-PBE-P86, respectively. The performance of most of the LC-DH functionals is also acceptable. The aforementioned functionals are more reliable than the fifth-order scaling wave function-based methods. The best global DH is the PBE0-2 approach, with a MAE of 0.39 eV, while somewhat higher errors are obtained for PBE-QIDH and its spin-scaled variants. Surprisingly, the error is fairly moderate for ωB97X, with a MAE of 0.26 eV. This performance is very close to those of the best RS-DH and LC-DH functionals. Unfortunately, the error is significantly higher for the others in this class, as the MAE is around 0.45 eV for the ωB97X-D and CAM-B3LYP methods. On the basis of these results, we can conclude that a highly similar order can be determined within the classes as for the previous intermolecular CT benchmark set. However, the absolute performance of the (SOS-)ωB2GPPLYP and ωB97X approaches is somewhat unexpected.

Interestingly, except for PBE0-2, the excitation energies are slightly overestimated for the best performers of the classes. The MEs are 0.05 and 0.11 eV for the RS-PBE-P86/SOS-ADC(2) and SOS-ωB2GPPLYP approaches, respectively, while a noticeably higher value is obtained for ωB97X. In this regard, the performance of the RS-DH and LC-DH functionals, apart from a few exceptions, is well-balanced. All of the global DH methods underestimate the excitation energies. The lowest ME of the class, precisely −0.31 eV, is achieved by PBE0-2. Similarly to the ME values, the MAX values are also fairly well-balanced for the RS-DH and LC-DH approaches. The maximum error is 0.52 eV for RS-PBE-P86/SOS-ADC(2), while a somewhat higher value is obtained for SOS-ωB2GPPLYP. For these classes, the lowest MAX is around 0.40 eV. In general, the maximum errors are a bit higher for the global DHs, but they are also well-balanced. The best performance is achieved by PBE0-2 with a MAX of 0.58 eV, whereas it exceeds 0.75 eV only for B2GPPLYP. A similar trend was observed for the LC hybrid methods as well. The MAX is 0.76 eV for the ωB97X functional. The lowest value, 0.62 eV, is attained by ωB97X-D, while the CAMh-B3LYP approach is inferior with a MAX of 1.03 eV.

### 4.3 A Brief Study on Other Types of Excitations

The main scope of this paper is to test the reliability of the most advanced TDDFT approaches for CT excitations. However, their performance for other types of transitions is also important. Accordingly, despite the excellent benchmark studies presented in the literature,^26,50,53−55^ a brief comparison is also carried out herein. In this case, the first benchmark set^124^ from the QUEST database^56^ proposed by Loos, Jacquemin, and co-workers is assessed. This well-balanced compilation, which is hereafter denoted as the LJ1 set, contains 52 singlet (25 valence and 27 Rydberg) “safe” values for small organic molecules, and high-quality TBE/aug-cc-pVTZ^125^ excitation energies are considered as the reference. The MAEs obtained for this compilation using the same basis sets in comparison with the CT results are collected in Table 2. The outcomes are discussed in detail only for the best performers in each class. As can be seen, for the LJ1 benchmark set, the performance of CCSD is outstanding, while the MAEs for the CT excitations are noticeably higher. It should be noted that the LJ1 compilation contains only small molecules and that the CCSD results are seriously affected by the system size.^107^ The poor performance of ADC(2) and CIS(D) for Rydberg excitations is well-known.^105^ Thus, these MAEs are comparable to the challenging intermolecular CT results. The values for valence excitations are more favorable, while the ADC(2) approach is also recommended for intramolecular CT transitions. Similar findings can be obtained for the spin-scaled ADC(2)-based RS-DH functionals, but all of the MAEs are significantly better in these cases, while the CIS(D)-based variants are less satisfactory for intramolecular CT excitations. For the spin-scaled ωPBEPP86 functionals, the accuracy is outstanding for the LJ1 test set, while the MAEs for the intramolecular CT transitions are comparable to the valence results. However, as revealed above, they are not recommended for intermolecular CT excitations. For the LJ1 benchmark set, fairly similar MAEs are achieved by the spin-scaled ωB88PP86 and ωB2GPPLYP approaches, but their accuracy for intramolecular CT transitions is closer to the somewhat less favorable Rydberg results. Inspecting the spin-scaled global DH functionals, we can conclude that the SCS/SOS-PBE-QIDH results are more balanced than those for DSD-PBEP86. That is, the differences between the valence and Rydberg errors are less considerable for the former functionals. In this class, the performance for the intramolecular CT excitations is closer to the valence results, but the deviation is higher for DSD-PBEP86. For the PBE0-2 and PBE-QIDH functionals, somewhat less favorable MAEs are obtained for valence transitions compared with the previous approaches, but the errors are highly acceptable. In addition, their performance is well-balanced, as significant differences between the intramolecular CT, valence, and Rydberg results cannot be found. The range-separated hybrids are inferior for valence excitations, while the outstanding accuracy of ωB97X for Rydberg transitions is fairly surprising. In the case of CAM-B3LYP, the MAE for intramolecular CT excitations is identical to the valence error, while it is significantly higher for ωB97X.

**Table 2:** MAEs for Various Types of Excitations

	CT		LJ1^124^	
method	intra.^57^	inter.^62^	valence	Rydberg
CCSD	0.30	0.30	0.08	0.08
ADC(2)	0.16	0.37	0.14	0.31
CIS(D)	0.35	0.37	0.21	0.36
RS-PBE-P86/SCS-ADC(2)	0.09	0.30	0.11	0.21
RS-PBE-P86/SOS-ADC(2)	0.12	0.22	0.10	0.21
RS-PBE-P86/SCS-CIS(D)	0.25	0.27	0.11	0.23
RS-PBE-P86/SOS-CIS(D)	0.24	0.24	0.13	0.23
SCS-ωPBEPP86	0.11	0.78	0.12	0.20
SOS-ωPBEPP86	0.12	0.66	0.11	0.20
SCS-ωB88PP86	0.21	0.63	0.12	0.26
SOS-ωB88PP86	0.18	0.64	0.11	0.26
SCS-ωB2GPPLYP	0.21	0.44	0.12	0.21
SOS-ωB2GPPLYP	0.25	0.36	0.12	0.20
SOS-RSX-QIDH	0.60	0.30	0.26	0.44
SOS-RSX-QIDH2	0.18	0.45	0.14	0.23
RSX-QIDH	0.50	0.33	0.24	0.35
ωB2PLYP	0.37	0.51	0.17	0.17
ωB2GPPLYP	0.37	0.38	0.17	0.19
SCS-PBE-QIDH	0.13	1.00	0.13	0.21
SOS-PBE-QIDH	0.12	0.98	0.12	0.21
DSD-PBEP86	0.16	1.08	0.11	0.25
PBE0-2	0.22	0.66	0.16	0.23
PBE-QIDH	0.18	0.97	0.18	0.20
B2GPPLYP	0.21	1.34	0.14	0.29
CAM-B3LYP	0.23	1.62	0.23	0.35
CAMh-B3LYP	0.35	2.03	0.25	0.47
ωB97X	0.36	0.99	0.21	0.14
ωB97X-D	0.18	1.69	0.22	0.29

## 5 Conclusions

The performance of the most recommended TDDFT functionals has been comprehensively tested for the recently proposed CT benchmark sets. For the detailed comparison, the state-of-the-art RS-DH and LC-DH methods including spin-scaling techniques were selected, and robust and popular global DH and LC hybrid approaches were also included in this study. Most of the functionals were developed to remedy the wrong long-range behavior of the XC energy. The overall performance of the methods is well-known from the relevant papers,^50,51,54^ but they have not yet been extensively investigated for CT excitations, which represent one of the most challenging problems for TDDFT approaches.

The functionals have been benchmarked on up-to-date test sets, such as the intramolecular CT compilation of Loos, Jacquemin, and co-workers^57^ and the benchmark set of Szalay et al.^62^ containing intermolecular CT transitions. In addition, the Ar–TCNE complexes proposed by Baer and co-workers^67^ were also assessed. Two of the compilations contain only high-quality CC-based reference values, while a short comparison was also presented with experimental results. The effects of the hole–particle distance using different CT metrics have been examined, and the correct long-range behavior of the XC energy has also been tested.

Our numerical results show that the most robust performances are attained by the ADC(2)-based RS-DH approaches. Only these functionals are suitable to describe both types of CT excitation with outstanding accuracy. The proposed RS-PBE-P86/SOS-ADC(2) approach^51^ is superior for intermolecular transitions, while practically only its SCS counterpart outperforms it for intramolecular excitations. Surprisingly, concerning the latter type of transitions, excellent results are obtained for the recently proposed spin-scaled global DH methods, such as SCS/SOS-PBE-QIDH.^54^ In other words, the results suggest that range separation is not necessary even for strong intramolecular CT excitations. In contrast, all of the global DH functionals failed for challenging intermolecular CT transitions, while serious limitations are also pointed out for the most recent LC-DH approaches. Despite the excellent performance of SCS- and SOS-ωPBEPP86^54^ for intramolecular excitations, they are inferior in describing transitions between distant parts of molecular complexes. Moreover, the wrong long-range behavior of the XC energy evaluated by these approaches is also demonstrated. The performance of the LC hybrid functionals is far from what was expected, as they cannot compete with the DH methods for either type of excitations.

## Supporting Information Available

Computed excitation energies, charge-transfer metrics, and fragmentation of the molecules (XLSX)
